# The regulatory roles of flavonoids, melatonin, and glycine betaine in plant salt stress response

**DOI:** 10.3389/fpls.2026.1774962

**Published:** 2026-05-08

**Authors:** Wenrong Yang, Zhaohui Jiang, Junwen Kong, Yanxiu Zhao, Shuangshuang Zhao

**Affiliations:** Shandong Provincial Key Laboratory of Plant Stress Biology and Genetic Improvement, College of Life Sciences, Shandong Normal University, Jinan, China

**Keywords:** biosynthesis, plant tolerance, regulatory network, salt stress, secondary metabolites

## Abstract

Salt stress, one of the most detrimental environmental constraints, severely impairs plant growth and productivity by inducing ionic imbalance, osmotic stress, and oxidative damage. In response, plants synthesize a diverse array of secondary metabolites that help maintain cellular turgor, improve water uptake, restore osmotic homeostasis, scavenge reactive oxygen species, and fine-tune growth and development—collectively enhancing salt tolerance. Although considerable progress has been made in characterizing individual metabolites, the intricate interplay between the regulation of secondary metabolism and plant salt stress adaptation remains poorly understood. This review summarizes recent advances in understanding how secondary metabolites respond to salt stress, with a particular focus on flavonoids, melatonin, and glycine betaine. We highlight emerging mechanisms governing their biosynthesis and accumulation under saline conditions and discuss the regulatory networks that coordinate their functions. Finally, we identify critical knowledge gaps and propose future research directions aimed at elucidating the interaction networks among these key secondary metabolites and their upstream regulatory hubs, with the goal of breeding crops with broad-spectrum stress resistance.

## 1 Introduction

Abiotic stresses pose significant threats to agricultural production worldwide, with salt stress being one of the most detrimental factors (Gong et al., 2020; Ma et al., 2025). High salinity restricts land usability and severely reduces crop yields by impairing plant growth and development (Su et al., 2020; Martínez Rivas et al., 2025). Salt stress induces a combination of primary and secondary stresses, including ion toxicity, osmotic stress, and oxidative damage (Yang and Guo, 2018a). These stresses collectively inhibit water uptake, disrupt cellular homeostasis, and impair photosynthesis (Yang and Guo, 2018b). In response, plants have evolved various adaptive mechanisms to maintain homeostasis and sustain growth under saline conditions. These strategies include osmotic adjustment, regulation of ion transport, and scavenging of reactive oxygen species (ROS) (Zelm et al., 2020; Zhao et al., 2021). Among these, metabolic regulation plays a particularly critical and dynamic role, enabling plants to undergo biochemical adjustments that support survival in harsh environments (Chapman and Muday, 2021).

Plants biosynthesize a diverse array of low-molecular-weight metabolites that are essential for growth regulation, defense, and environmental adaptation (Zhao et al., 2019; Zhou et al., 2019; Maeda, 2019). These compounds are broadly categorized into primary metabolites, which sustain fundamental cellular processes; phytohormones, which coordinate development and stress signaling; and secondary metabolites, which primarily mediate plant-environment interactions (Erb and Kliebenstein, 2020). Importantly, the functional repertoire of key secondary metabolites extends beyond salt stress, serving as conserved mechanisms for abiotic stress tolerance more broadly. For instance, exogenous melatonin has been shown to enhance drought resilience by improving physiological traits and reprogramming stress-related metabolic pathways in species such as *Taxus baccata* and *Prunus avium* (Shahmohammadi et al., 2024; Hojjati et al., 2024). Similarly, glycine betaine application mitigates water deficit by supporting membrane integrity and biochemical functions (Annunziata et al., 2019). This findings underscore the fundamental role of these metabolites as broad-spectrum stress regulators, with this review focusing on their specific functions under saline conditions.

Secondary metabolites comprising more than 200,000 structurally diverse compounds, are derived from primary metabolic pathways and include nitrogen-containing compounds, fatty acid derivatives, phenolics, alkaloids, terpenoids, benzenoids, indole and sulfur-containing indole compounds, and glucosinolates (Piasecka et al., 2015). These compounds are particularly significant in mediating plant responses to environmental cues, including salt stress (Yang et al., 2018). Their biosynthesis and accumulation are often triggered by specific stress conditions through changes in gene expression and enzymatic activity (Li et al., 2020).

**Figure 1:**
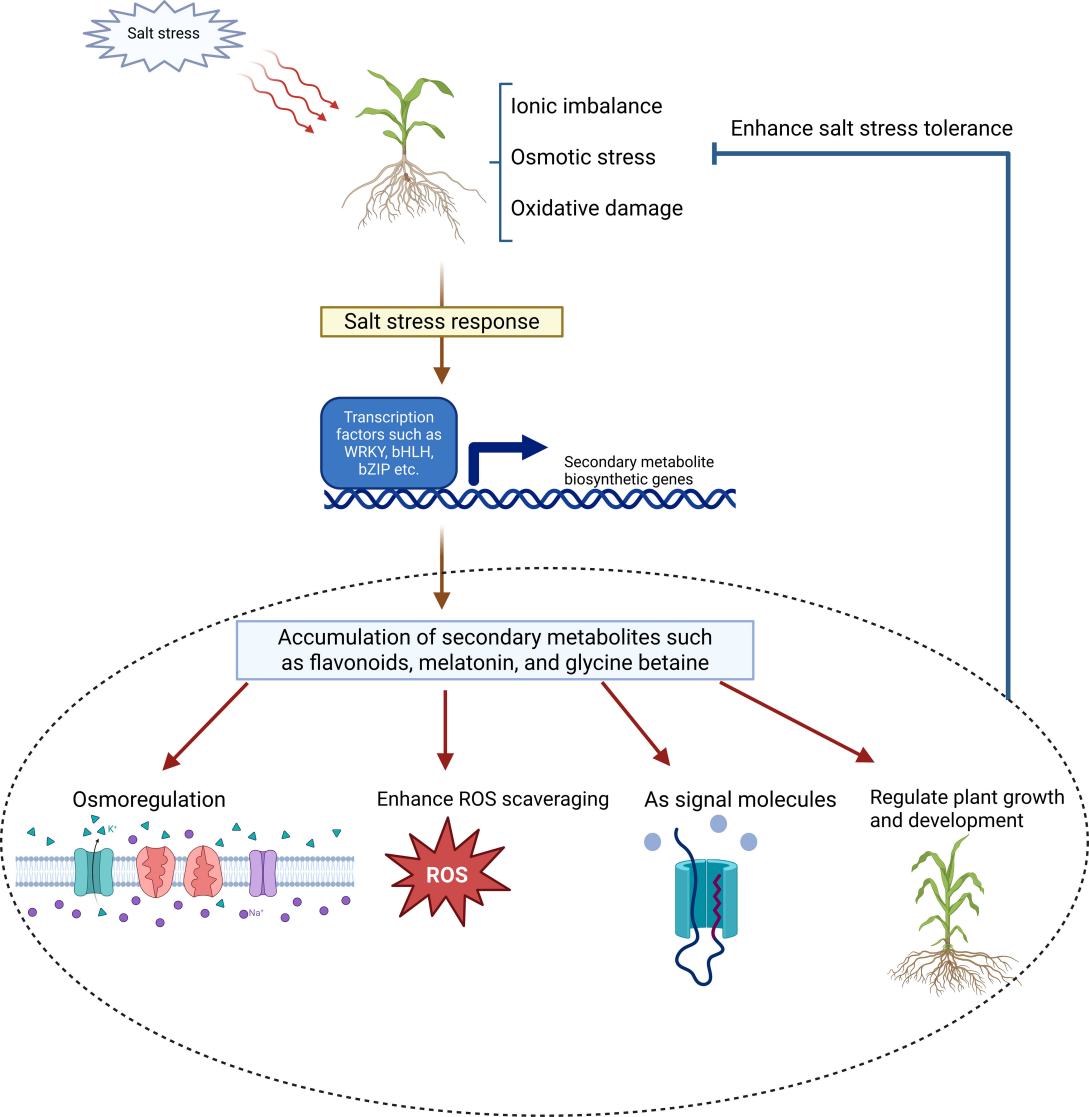
Signaling and metabolic pathway activated by salt stress in plants. Salt stress triggers ionic imbalance, osmotic stress, and oxidative damage, which collectively activate the salt stress response. This leads to the upregulation of transcription factors (e.g., WRKY, bHLH, bZIP), which in turn induce the expression of secondary metabolite biosynthetic genes. The resulting accumulation of key secondary metabolites (e.g., flavonoids, melatonin, and glycine betaine) mediates multiple adaptive responses, including osmoregulation, enhanced ROS scavenging, signaling, and the regulation of plant growth and development, ultimately alleviating salt-induced damage.

In the face of high salinity, plant secondary metabolic processes are activated to ensure survival (Nogia and Pati, 2021). Secondary metabolites help in adapting to osmotic stress by acting as organic osmolytes, regulating cellular turgor, and facilitating water uptake (Ghosh et al., 2021). They also mitigate ion stress by modulating ion channel activity, maintaining intracellular ionic balance and protecting against sodium toxicity (Yang and Guo, 2018b). Furthermore, elevated salt stress leads to a rise in ROS levels, imposing oxidative stress. Accumulated secondary metabolites such as phenolics, flavonoids, and alkaloids help neutralize ROS, thereby restoring redox homeostasis and protecting cellular structures (Zhang et al., 2025a; Pastacaldi et al., 2024; Li et al., 2020) (**Figure 1**).

Despite these advances, the molecular mechanisms governing the biosynthesis and functional roles of secondary metabolites under salt stress remain largely unexplored. Elucidating these regulatory networks is essential for understanding how plants dynamically reprogram their metabolism to enhance stress tolerance and adapt to changing environments (Yang et al., 2018; Olmos et al., 2024). Among the diverse array of secondary metabolites, flavonoids, betaine, and melatonin represent three typical and chemically distinct classes (phenolics, indoleamines, and quaternary ammonium compounds), exhibiting distinct physiological functions in salt stress responses (osmotic adjustment, oxidative scavenging, and signal transduction) (Petrussa et al., 2013; Annunziata et al., 2019; Zhao et al., 2019). Their inherent metabolic interconnections contribute to a multilayered defense network against salinity (Khalid et al., 2022). This review synthesizes current research on these three metabolite groups, with a focus on their unique contributions and potential synergistic interactions in improving plant salt tolerance. A deeper understanding of these mechanisms will be instrumental for harnessing metabolic pathways to enhance crop productivity and develop salt-tolerant plant varieties.

## 2 Role of secondary metabolites in the plant salt stress response

Under salt stress, the secondary metabolites accumulated by plants constitute a crucial line of defense, functioning as osmolytes to regulate osmotic balance, as antioxidants to mitigate oxidative stress, as signal molecules to activate signaling pathways, or as growth regulators to directly modulate plant development.

### 2.1 Secondary metabolites serve as osmolytes involved in osmoregulation

High salinity leads to excess Na^+^ accumulation in the vacuole, which alters the cell cytosolic turgor pressure, limits water uptake and induces osmotic stress. As a general response, plants produce a series of compatible cytosolic solutes to regulate the osmotic imbalance (Ghosh et al., 2021). Secondary metabolites such as glycine betaine, alkaloids, flavonoids, and terpenoids play prominent roles in osmoregulation (Buer et al., 2010; Yang and Guo, 2018a; Dabravolski and Isayenkov, 2023) (**Figure 1**). These compounds have low molecular weights and are highly compatible with the cellular biochemical processes, enabling them to stabilize cellular structures, mitigate the harmful effects of Na^+^ accumulation, and enhance water retention. For instance, in *Stevia rebaudiana* glycine betaine not only regulates intracellular osmotic potential, but also maintains K^+^/Na^+^ balance by decreasing vacuolar Na^+^ accumulation while increasing cytosolic K^+^ absorption (Ghorbani et al., 2023a).

### 2.2 Secondary metabolites function as antioxidants in ROS scavenging

Salt stress triggers oxidative stress, leading to the accumulation of ROS such as hydroxyl radicals (OH•), superoxide radicals (O_2_^•−^), singlet oxygen (¹O_2_), and hydrogen peroxide (H_2_O_2_). If not effectively removed, excess ROS can disrupt cellular homeostasis and ultimately result in cell death (Zhao et al., 2021; Wang et al., 2024a). To mitigate oxidative stress, plants activate both enzymatic and non-enzymatic antioxidant systems, the latter of which includes a variety of secondary metabolites (Chapman and Muday, 2021). The enzymatic scavenging system comprises ROS-scavenging enzymes such as superoxide dismutase (SOD), catalase (CAT), glutathione S-transferase (GST), ascorbate peroxidase (APX), and glutathione peroxidase (GPX) (Su et al., 2020; Zhang et al., 2025b). In contrast, non-enzymatic defense relies on secondary metabolites—including phenolics, flavonoids, glycine betaine and other alkaloids to quench ROS (Yang and Guo, 2018b; Dvořák et al., 2021).

Numerous secondary metabolites involved in the non-enzymatic antioxidant system have demonstrated significant ROS-scavenging capacity *in vitro* (Erb and Kliebenstein, 2020). Under saline conditions, plants accumulate elevated levels of phenolic compounds (e.g., isoorientin, rutin, vitexin, vanillin, lignin, and protocatechuic acid), terpenoids (e.g., carotenoids, oleuropein, cineole, and camphene), alkaloids (e.g., betaine, choline, and catharanthine), and flavonoids (e.g., anthocyanins). These metabolites play essential roles in neutralizing ROS and protecting cellular structures (Borghesi et al., 2011; Yahyazadeh et al., 2018; Chun et al., 2019) (**Figure 1**).

### 2.3 Secondary metabolites as signal molecules

Under high salinity, plants perceive stress stimuli such as Na^+^ accumulation, leading to calcium oscillation and increased ROS levels (Zelm et al., 2020). The calcium and ROS signals serve as key messengers to activate the salt stress signaling pathways, which trigger downstream physiological responses (Zhao et al., 2021). Secondary metabolites play an essential role in signal transduction, acting as bioactive molecules that orchestrate these stress-adaptive pathways (Yang and Guo, 2018a). One prominent example is melatonin, which regulates multiple downstream signaling pathways, including interactions with hormones, nitric oxide (NO), and polyamine metabolism (Zhan et al., 2019). Melatonin has been shown to influence the biosynthesis and metabolism of abscisic acids (ABA), reducing its accumulation to mitigate the adverse effects of salt stress (Tan et al., 2021). In addition, melatonin interacts synergistically with NO to enhance stress tolerance. For instance, in rapeseed seedlings, NO improves salt tolerance by maintaining ion homeostasis and amplifies melatonin’s role in ROS detoxification (Zhao et al., 2018). Furthermore, melatonin plays a crucial role in polyamine-mediated signaling pathways under diverse abiotic stress conditions (Zhao et al., 2017). It enhances polyamine biosynthesis in wheat seedlings by accelerating the conversion of arginine and methionine into polyamines such as spermidine (Spd) and spermine (Spm). Additionally, melatonin inhibits the activity of polyamine oxidase (PAO) and diamine oxidase (DAO), two enzymes involved in polyamine metabolism, thereby sustaining higher polyamine levels and improving plant salt tolerance (Ke et al., 2018). Altogether, secondary metabolites such as melatonin function as sophisticated signaling molecules that modulate diverse stress-related pathways, enabling plants to maintain ion balance, mitigate oxidative stress, and enhance overall resilience to salinity (**Figure 1**).

### 2.4 Secondary metabolites directly regulate plant growth and development

Secondary metabolites can also directly modulate plant growth and development in response to salt stress (**Figure 1**). For instance, polyamines are widely recognized for their involvement in growth regulation and stress adaptation (Masson et al., 2017). The primary polyamines—putrescine, spermidine and spermine, influence diverse aspects of plant development including organogenesis, embryo maturation, floral initiation and development, leaf senescence, fruit development and maturation (Wimalasekera et al., 2011). These polyamines not only play pivotal roles in normal growth but also improve stress tolerance by stabilizing cellular structures and modulating gene expression during abiotic and biotic stress responses. Exogenous application of polyamines or manipulation of endogenous polyamine levels has been shown to enhance stress resistance and promote plant development under salt stress (Sánchez-Rodríguez et al., 2016; Blázquez, 2024).

Beyond polyamines, steroids serve critical functions as precursors for key phytohormones such as ABA and brassinosteroids, which play important roles in growth regulation under salt stress. These phytohormones coordinate responses to environmental stressors by controlling processes such as stomatal closure, cellular expansion, and the regulation of flowering and senescence (Erb and Kliebenstein, 2020). For example, ABA mediates water retention and root growth during salinity-induced drought-like conditions, while brassinosteroids influence cell elongation and division, ensuring normal development under challenging environments (Isah, 2019).

In addition to the previously discussed metabolites, flavonoids, melatonin, and glycine betaine also play direct and pleiotropic roles in regulating plant growth and development under both optimal and stress conditions. Flavonoids modulate auxin transport by inhibiting specific PIN-FORMED (PIN) efflux carriers, thereby influencing root architecture, tropic responses, and shoot development. They also affect pollen germination and tube growth, which are critical for successful fertilization (Santelia et al., 2008; Peer et al., 2004; Zhang et al., 2021a). Melatonin functions as a growth regulator that promotes seed germination, root regeneration, and leaf senescence delay. It interacts with auxin and gibberellin signaling pathways to modulate cell division and expansion. Additionally, melatonin regulates flowering time and acts as an antioxidant to protect developing tissues from oxidative damage (Lv et al., 2021; Mukherjee, 2018; Khalid et al., 2022). Glycine betaine also directly impacts growth and development. It can enhance photosynthetic efficiency by protecting thylakoid membranes and Rubisco activity, leading to increased biomass accumulation. It also promotes root elongation and branching under non-stress conditions and supports reproductive development by improving pollen viability and seed set (Khalid et al., 2022; Kulak and Gulmez, 2023). Collectively, these examples underscore that flavonoids, melatonin, and glycine betaine are integral regulators of plant growth and developmental programs.

## 3 Secondary metabolites regulated by salt stress

### 3.1 Flavonoids

**Figure 2:**
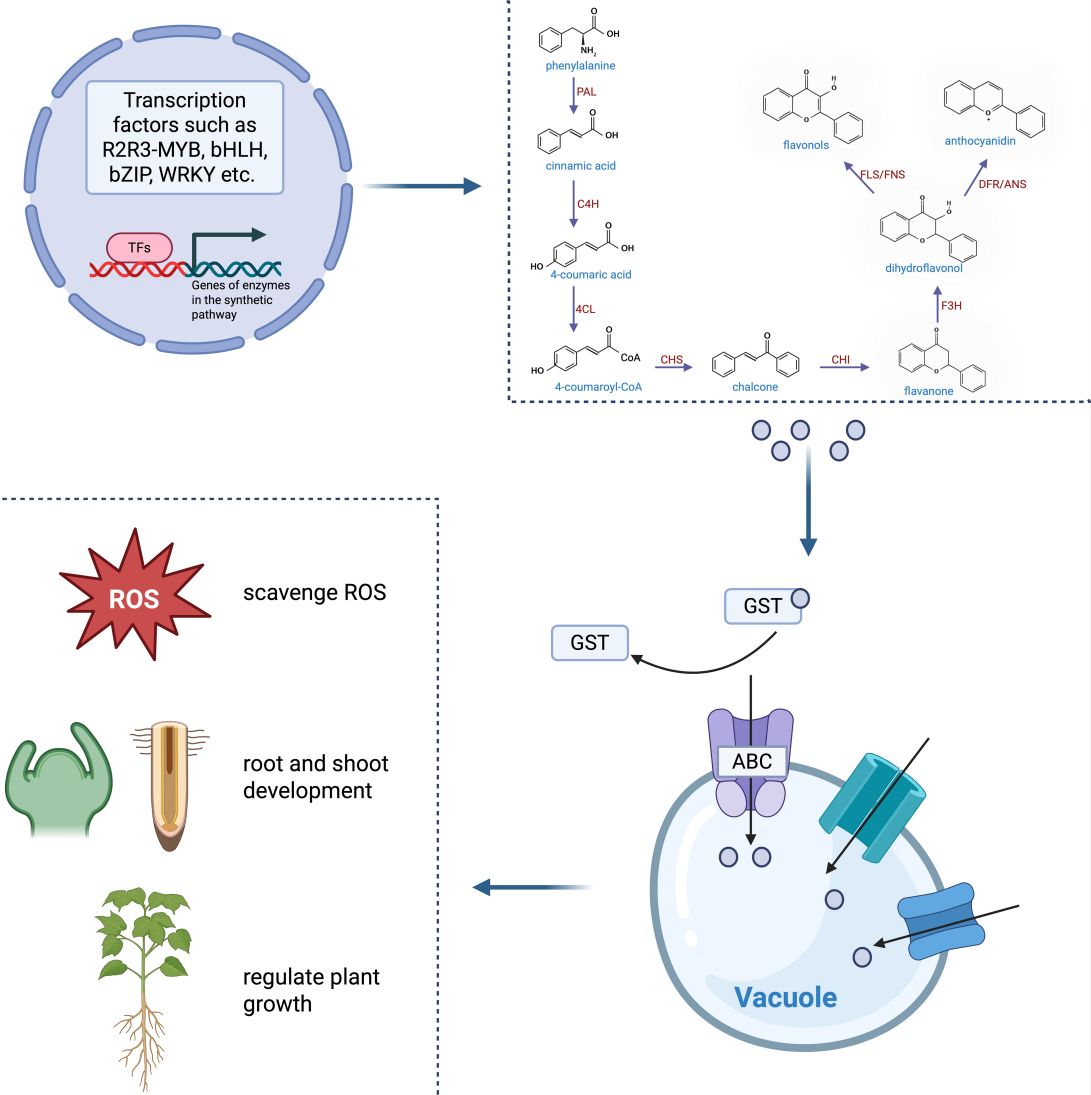
Regulation and function of flavonoid biosynthesis in plants. The biosynthesis of flavonoids is transcriptionally regulated by a suite of transcription factors (TFs), including R2R3-MYBs, bHLHs, bZIPs, and WRKYs. These TFs activate genes encoding key enzymes in the flavonoid biosynthetic pathway. Synthesized flavonoids are transported into vacuoles via ABC transporters, MATE transporters, and glutathione S-transferase (GST)-mediated mechanisms. These flavonoids respond to salt stress by scavenging ROS, promoting the growth of roots and shoots, and regulating plant growth.

Flavonoids, a major class of polyphenolic secondary metabolites derived from phenylpropanoid metabolism, are widely distributed in higher plants (Wang et al., 2025b). Their biosynthesis begins with phenylalanine, which is converted through a series of enzymatic reactions into chalcone, a key intermediate (Liu et al., 2021) (**Figure 2**). Chalcone is subsequently isomerized to flavanone, from which the pathway branches to produce diverse flavonoid classes through distinct enzyme cascades. Flavanone 3-hydrogenase (F3H) converts flavanone into dihydroflavone, which is then directed toward flavonols by flavonol synthetase (FLS) or toward flavones by flavone synthetase (FNS). In anthocyanin synthesis, dihydroflavone is reduced to leucoanthocyanidin by dihydroflavone 4-reductase (DFR), and subsequently oxidized to anthocyanins by anthocyanin synthetase (ANS) (**Figure 2**; **Table 1**).

**Table 1:** Roles of flavonoids, melatonin, and glycine betaine in plant salt stress responses.

Representative metabolites	Class of secondary metabolites	Key structural genes	Mode of action	Representative species	Reference
Flavonoids	Phenolics	F3H/F3’H, FLS/FNS, DFR/ANS	scavenging ROS, reducing membrane lipid peroxidation, osmoregulation, activating the expression of stress-responsive genes; hormone interaction	*Oryza sativa*, *Glycine max*, *Limonium bicolor*	Davies et al, 2024; Ni et al., 2025; Wang et al., 2025a; Wu et al., 2025; Zhu et al., 2025
Melatonin	Indoleamines	TDC, T5H, SNAT, COMT/ASMT	scavenging ROS, maintaining ion homeostasis, osmoregulation, photosynthesis protection, hormonal regulation, activating MAPK cascades, influencing metabolites accumulation	*Solanum lycopersicum, Triticum aestivum, Malus domestica*	Liu et al., 2019; Shamloo-Dashtpagerdi et al., 2022; Hu et al., 2025; Michalek et al., 2025
Glycine betaine	Alkaloids	CMO, BADH	scavenging ROS, maintaining ion homeostasis, osmoregulation, protein stabilization, reduce transpiration, modulating C/N balance	*Arabidopsis thaliana*, *Oryza sativa*, *Nicotiana tabacum, Triticum aestivum*	Yang, et al., 2008; Hasanuzzaman et al., 2014; Wei et al., 2017; Sofy et al., 2020

Under salt stress, this biosynthesis network is strongly upregulated, resulting in increased accumulation of flavonoids to enhance plant tolerance (Davies et al., 2024; Liu et al., 2021). For example, structural genes such as *F3H* and *DFR* are induced by salt stress and their overexpression has been shown to elevate flavan-3-ol or anthocyanin content, and improve salt tolerance in transgenic plants (Mahajan and Yadav, 2014; Kim et al., 2017). Transcriptional regulation plays a central role in modulating flavonoid synthesis under salt stress. Several transcription factor families such as MYB, bHLH, WRKY, NAC, and bZIP are key regulators of flavonoid biosynthesis genes (Chen et al., 2019; Zheng et al., 2025). Among them, MYB transcription factors such as in strawberry *FvMYB24*, and in alfalfa *MsMYB206*, are particularly important in mediating salt-induced flavonoid accumulation (Wang et al., 2021; Su et al., 2025). Additionally, the regulatory module LncNAT11–GbMYB11–GbF3’H/GbFLS identified in *Ginkgo biloba*, has been identified as a critical determinant of flavonoids biosynthesis under salt stress. In this module, GbMYB11 directly binds to the promoters of *GbF3’H* and *GbFLS*, enhancing their transcription and promoting flavonoids production. Conversely, the antisense lncRNA *LncNAT11* represses *GbMYB11* expression and is downregulated by salt stress relieving this repression (Liu et al., 2025). In summary, the flavonoids biosynthetic pathway is markedly activated under salt stress. Key enzymes (e.g., F3H/F3’H, FLS, and DFR) and transcriptional regulatory modules collectively promoting the accumulation of flavonoids, thereby systematically strengthening plant adaptation to salt stress (**Figure 2**). In addition to the biosynthesis network, the transport and subcellular compartmentalization of flavonoids are also subject to intricate regulation (Nguyen et al., 2014; Du et al., 2021; Niu et al., 2023). These processes are critical for their stress-responsive functions (Yang et al., 2018; Chapman and Muday, 2021). Flavonoids are translocated to the apoplast or vacuoles via ATP-binding cassette (ABC) transporters, multidrug and toxic compound extrusion (MATE) proteins, and glutathione S-transferase (GST)-mediated mechanisms (Kim et al., 2010; Petrussa et al., 2013; Pastacaldi et al., 2024) (**Figure 2**).

Flavonoids play multifaceted protective roles in plant salt tolerance, with their antioxidant function being the most prominent. By donating hydrogen atoms from phenolic hydroxyl groups, flavonoids can bind peroxyl radicals and directly scavenge oxygen radicals, functioning as potent non-enzymatic antioxidants (Zhou et al., 2023). This activity reduces lipid peroxidation and preserves plasma membrane integrity, thereby enhancing overall salt tolerance (Khalid et al., 2022). For example, the halophyte *Limonium bicolor* accumulates flavonoids under saline conditions to alleviate oxidative stress (Zhu et al., 2025). Similarly, salt-tolerant plants often accumulate flavonoids such as quercetin and kaempferol to boost antioxidant capacity and cope with saline environments (Xu et al., 2020).

In addition to their antioxidant roles, flavonoids contribute to osmotic regulation under salt stress. They promote the accumulation of compatible solutes such as proline and soluble sugars, which lower osmotic potential, facilitate water uptake, and help maintain cellular osmotic balance under high-salinity conditions (Xu et al., 2020; Zhou et al., 2023; Zhu et al., 2025).

Moreover, flavonoids may function as signaling molecules that induce the expression of salt-tolerance-related genes, including high-affinity potassium transporters (HKT) and Salt Overly Sensitive (SOS) pathway genes, thereby initiating adaptive responses and reinforcing salt stress resilience. Beyond stress responses, flavonoids also regulate various aspects of plant growth and development, such as flowering, fruiting, and seed germination (Jan et al., 2021; Singh et al., 2021; Wang et al., 2024b).

Flavonoids further interact with phytohormones to coordinately modulate stress resistance mechanisms. Studies have shown that overexpression of the transcription factor *PRODUCTION OF ANTHOCYANIN PIGMENT 1 (PAP1)* promotes flavonoids biosynthesis while simultaneously altering phytohormone levels, indicating cross-talk between flavonoids metabolism and hormonal homeostasis (Zhang et al., 2025a). Phytohormones play crucial roles in regulating flavonoids biosynthesis: abscisic acid (ABA), ethylene, jasmonic acid (JA), cytokinins, and brassinosteroids generally enhance flavonoid production, whereas auxin suppresses it. For instance, ABA treatment in pigeon pea (*Cajanus cajan*) induces substantial accumulation of flavonoids metabolites.

In summary, flavonoids serve as essential metabolites and signaling molecules that enhance plant resilience to salt stress through antioxidative activity, osmoregulation, transcriptional reprogramming, and hormone interaction, making them a focal point in understanding plant adaptation to salt stress (**Table 1**).

### 3.2 Melatonin

**Figure 3:**
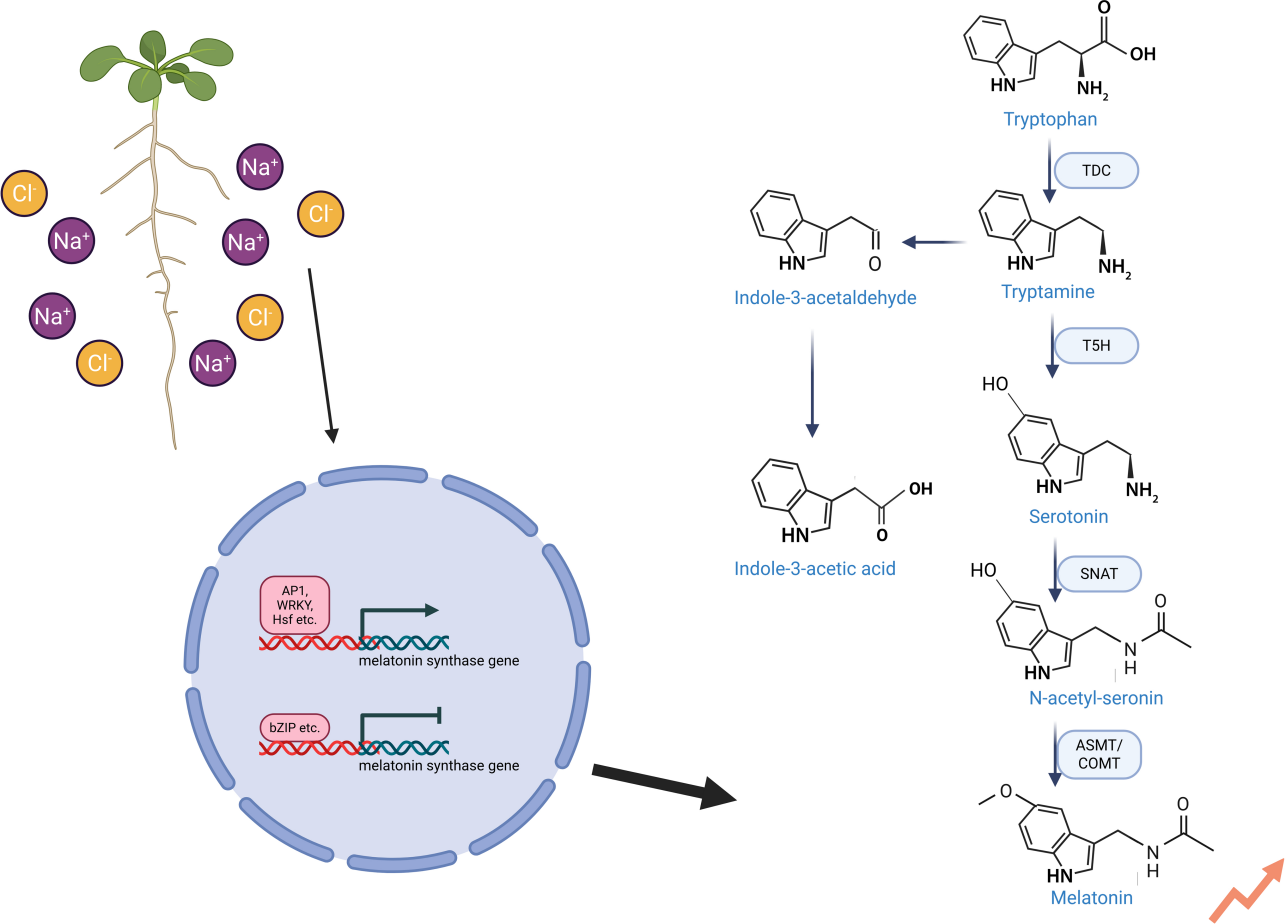
Regulation of melatonin biosynthesis under salt stress in plants. Under salt stress, transcription factors such as AP1, WRKY, and Hsf upregulate the expression of melatonin biosynthesis-related genes, enhancing the activity of key enzymes to promote melatonin production. Conversely, bZIP transcription factors suppress these genes, reducing melatonin synthesis. Melatonin shares functional similarities with the auxin indole-3-acetic acid (IAA), and both are derived from the common precursor tryptophan. The biosynthesis of melatonin begins with tryptophan, which is decarboxylated by TDC to form tryptamine, then hydroxylated to produce serotonin. Subsequent enzymatic reactions catalyzed by SNAT, ASMT, and COMT convert serotonin into melatonin. This coordinated regulation fine-tunes melatonin levels, aiding plant adaptation to saline conditions.

Melatonin (N-acetyl-5-methoxytryptamine) is a pleiotropic molecule in plants, integral to both biotic and abiotic stress responses. It exhibits functional similarities to auxin (indole-3-acetic acid, IAA) as both share the common biosynthetic precursor tryptophan (Li et al., 2019). In plants, tryptophan is decarboxylated to tryptamine, which is then hydroxylated to produce serotonin. Through enzymatic reactions catalyzed by serotonin N-acetyltransferase (SNAT), acetylserotonin O-methyltransferase (ASMT), and caffeic acid O-methyltransferase (COMT), serotonin is ultimately converted to melatonin (Zhao et al., 2019; Sun et al., 2021) (**Figure 3**; **Table 1**).Under normal growth conditions, melatonin is maintained at low concentrations; however, under salt stress, its biosynthesis increases rapidly (Khan et al., 2022). In cotton seedlings, salt stress triggers melatonin accumulation, which modulates antioxidant enzymes and coordinates plant hormones such as auxin, ABA, and BR, bolstering stress tolerance (Zhang et al., 2021b). Under high salinity, stress-responsive transcription factors such as AP-1 stimulate melatonin synthesis by upregulating melatonin biosynthesis-related genes (Zhao et al., 2019). In alfalfa, *SNAT1* overexpression enhances melatonin synthesis and salt tolerance, while bZIP55 negatively regulates its expression, reducing melatonin-mediated stress resilience (Wang et al., 2025b). In wheat, the O-methyltransferase 1 (OMT1) gene enhances melatonin production, aiding adaptation to salt stress (Shamloo-Dashtpagerdi et al., 2022). These findings demonstrate that plants activate melatonin biosynthesis pathways as an adaptive mechanism to cope with salt-induced stress. This regulatory capacity is not unique to salt stress; melatonin also dynamically upregulates key biosynthetic genes, such as those in the taxol pathway under drought conditions, highlighting its role as a master metabolic integrator (Shahmohammadi et al., 2024, 2025).

Salt stress reduces chlorophyll content, limits photosystem II (PSII) efficiency, and disrupts light absorption, electron transfer, and overall photosynthetic performance—a major factor in plant growth inhibition (Tal et al., 2011; Lazár et al., 2013). Melatonin mitigates these impacts by protecting chlorophyll from degradation, enhancing PSII reaction center efficiency, and upregulating photosynthesis-related genes, including *PsaK* and *PsaG* for PSI components and *PsbO* and *PsbP* for PSII subunits (Li et al., 2017). Salt stress also induces stomatal closure to reduce water loss, which lowers CO_2_ availability and photosynthetic rate (Brugnoli and Lauteri, 1991). Melatonin increases stomatal conductance, reopening stomata under stress via its receptor PMTR1-mediated signaling pathway (Wei et al., 2018). It also impacts glucose and fatty acid metabolism, alongside ABA synthesis, to sustain photosynthetic activity (Shi et al., 2015). Collectively, melatonin alleviates salt-induced photosynthetic inhibition by preserving chlorophyll integrity, enhancing PSII efficiency, upregulating photosystem gene expression, and modulating stomatal conductance, thereby maintaining photosynthetic performance under stress conditions.

**Figure 4:**
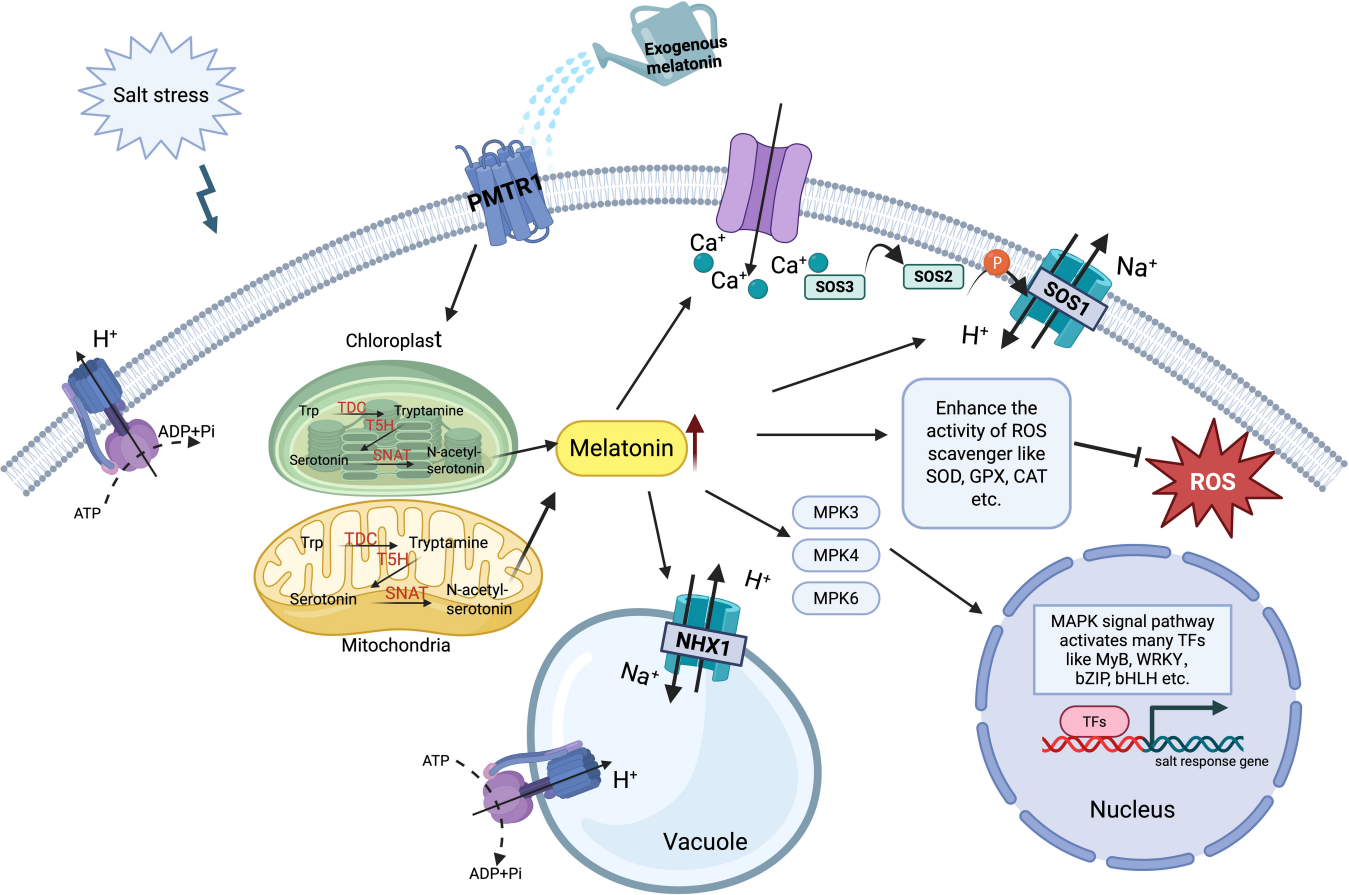
Mechanisms of melatonin-mediated salt stress tolerance in plants. Exogenous melatonin is perceived by the receptor PMTR1, which upregulates key enzymes in mitochondria and chloroplasts to enhance endogenous melatonin biosynthesis. Elevated melatonin levels activate: (1) Ion homeostasis. Ca^2+^ signaling (via SOS3) triggers the SOS pathway for Na^+^ extrusion, while *NHX1* facilitates vacuolar Na^+^ sequestration. (2) ROS scavenging. SOD, CAT, and GPX are activated to eliminate stress-induced ROS. (3) Transcriptional reprogramming. The MAPK cascade (MPK3/4/6) induces salt-responsive transcription factors (MYB, WRKY, bZIP, bHLH), boosting expression of stress-tolerance genes.

Melatonin functions as a potent antioxidant in plant responses to salt stress, coordinating the detoxification of ROS and reactive nitrogen species (RNS) to maintain cellular redox homeostasis. Its structure, composed of electron-rich indole groups and side chains (5-methoxy and 3-amide), provides strong free radical scavenging activity through mechanisms such as electron donation (forming melatoninyl action radicals) and hydrogen donation (nitrosation or substitution reactions) (Colak et al., 2024). Melatonin regulates antioxidant enzymes, including SOD, GPX, and CAT, to alleviate the toxic effects of ROS accumulation (Tal et al., 2011; Rodriguez et al., 2004). Additionally, exogenous melatonin induces the accumulation of polyphenols such as phenolic acids, flavonoids, and anthocyanins, which combat oxidative damage and improve plant salt tolerance (Colak et al., 2024). Melatonin also interacts with signaling molecules like NO to modulate redox balance. For example, in sunflower cotyledons, melatonin and NO jointly regulate glutathione (GSH) levels and glutathione reductase activity to protect cells from stress damage (Kaur and Bhatla, 2016). Furthermore, COMT-mediated melatonin synthesis reduces NO levels, preventing nitrosation damage to plasma membrane proteins like H^+^-ATPase 2 (HA2), thus improving salinity tolerance (Wei et al., 2025; Qin et al., 2023) (**Figure 4**). In conclusion, melatonin orchestrates a comprehensive antioxidant defense system against salt stress by directly scavenging ROS/RNS, modulating key antioxidant enzymes, inducing protective polyphenols, and interacting with signaling molecules such as NO to maintain redox homeostasis and mitigate oxidative damage.

Melatonin acts as a key regulator of ion homeostasis under salt stress. By promoting the expression of ion transport genes, melatonin helps maintain low cytoplasmic Na^+^ and high K^+^ levels, thereby alleviating ionic toxicity. Specifically, melatonin upregulates *NHX1* (Na^+^/H^+^ antiporter) and *AKT1* (K^+^ channel), while also enhancing the expression of *SOS1* for Na^+^ efflux and *CLC1/CLC2* for Cl^-^ transport (Ghorbani et al., 2023b; Wang et al., 2023; Khan et al., 2024). Through these mechanisms, melatonin facilitates vacuolar Na^+^ sequestration and cytoplasmic K^+^ uptake, ultimately strengthening plant tolerance to salinity. In addition, exogenous melatonin enhances ion homeostasis under salt stress by significantly increasing the activities of plasma membrane H^+^-ATPase and vacuolar (V)-H^+^-ATPase in cucumber leaves (Li et al., 2025a). These findings provide strong evidence that melatonin plays a critical role in sustaining ion balance and enhancing salt tolerance.

Melatonin interacts with diverse signaling cascades to bolster plant resilience under salt stress (Yu et al., 2018). Key pathways include phosphatidylinositol signaling, which mobilizes Ca^2+^ release to mediate stress responses; activation of the MAPK pathway (MPK3, MPK4, and MPK6) to drive stress-responsive gene expression; and regulation of lipid signaling pathways to preserve membrane integrity and maintain phospholipid balance under stress (Ke et al., 2018; Zhang et al., 2021b; Maity et al., 2022) (**Figure 4**). Additionally, melatonin enhances hormonal signaling by interacting with ethylene, JA, salicylic acid, and auxin to coordinate plant growth and development during stress (Pelagio-Flores et al., 2012; Arnao and Hernández-Ruiz, 2019). For instance, melatonin and auxin synergistically modulate root system architectures, improving adaptation to saline environments (Zhang et al., 2020). Collectively, melatonin enhances plant salt tolerance by orchestrating multiple signaling pathways and synergizing with hormonal networks such as auxin to optimize adaptive growth responses. A consolidated overview of melatonin’s diverse roles is provided in **Figure 4**.

Taken together, melatonin is a versatile signaling molecule and potent antioxidant that plays critical roles in stress adaptation by regulating ROS/RNS balance, improving photosynthetic efficiency, maintaining ion homeostasis, and activating multiple signaling pathways. The combination of melatonin’s direct biochemical functions and its interactions across intracellular pathways highlights its vital role in improving plant resilience in adverse environments (**Table 1**).

### 3.3 Glycine betaine

Glycine betaine is a zwitterionic quaternary ammonium compound naturally occurring in plants (Sharma et al., 2024). Classified as an important compatible solute, glycine betaine plays a crucial role in alleviating the adverse effects of salt stress on plant growth (Sofy et al., 2020). While halophytes naturally accumulate glycine betaine as an adaptive mechanism, only a subset of higher plants–such as those in the Poaceae, Portulaceae, Asteraceae, and Malvaceae families–synthesize and store this compound under stress conditions (Zhu et al., 2022).

**Figure 5:**
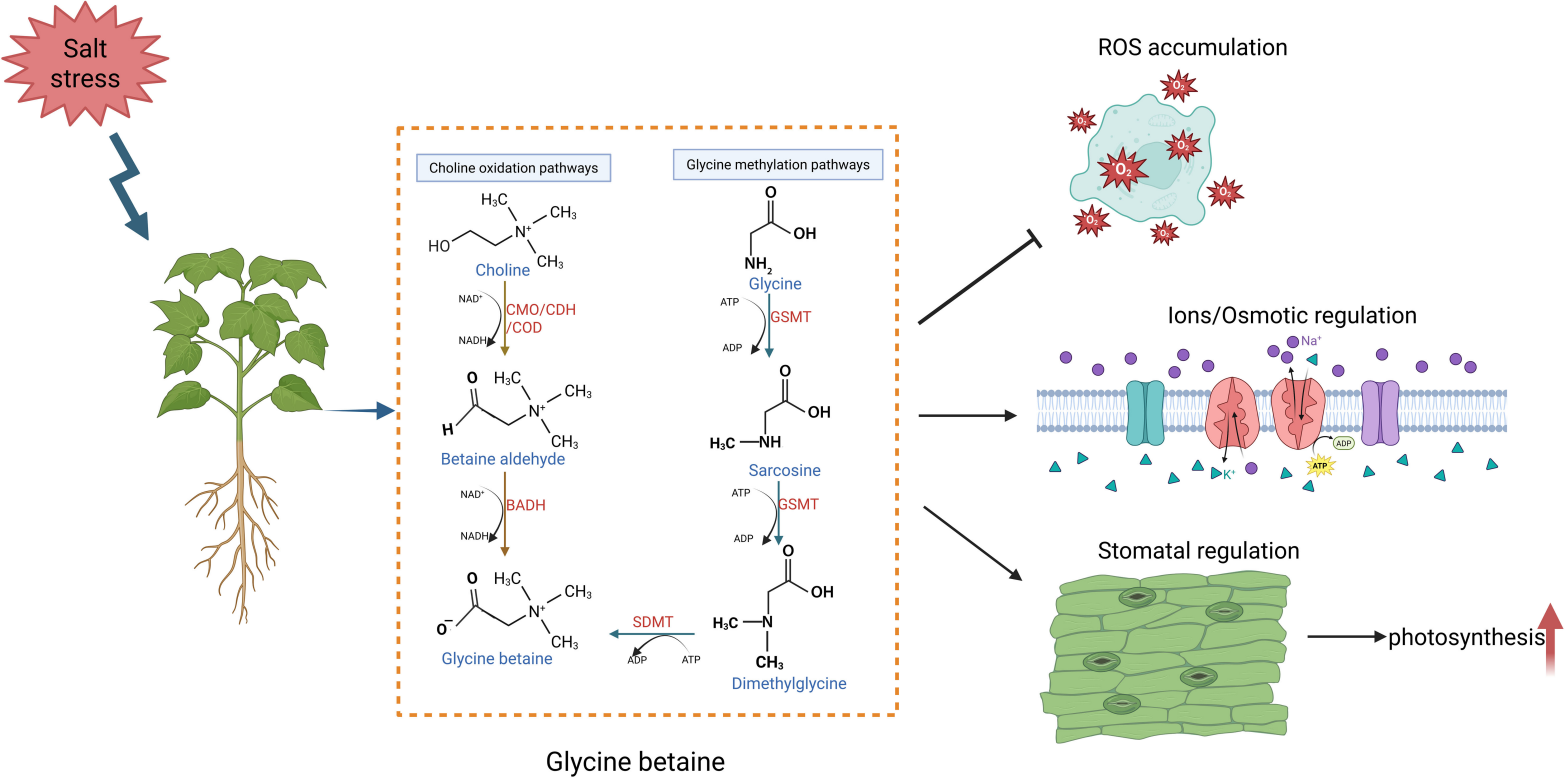
Glycine betaine enhances salt tolerance in plants under salt stress. Under saline conditions, plants accumulate higher levels of glycine betaine, which scavenges excess reactive oxygen species (ROS), regulates ion and osmotic homeostasis, and improves photosynthetic efficiency by modulating stomatal closure, thereby enhancing salt stress adaptation.

Glycine betaine is synthesized within chloroplasts through a two-step oxidation of choline, involving the enzymes choline monooxygenase (CMO) and betaine aldehyde dehydrogenase (BADH). These enzymes are tightly regulated under salt stress conditions (Sofy et al., 2020). For instance, salt stress enhances the expression of *CMO* and *BADH* genes, leading to increased enzyme activity and glycine betaine accumulation (Li et al., 2025b; Niazian et al., 2021). During salt stress, *CMO* expression significantly increases in the guard cells and roots of in *Beta vulgaris* (Rasouli et al., 2020). Tobacco plants ectopically expressing the *CMO* gene from Beta vulgaris exhibit enhanced salt tolerance through increased glycerol betaine levels (Zhang et al., 2008). Additionally, glycine betaine can be synthesized indirectly from serine via the non-phosphorylated glyceroic acid and glycolic acid pathways induced during salt stress (Annunziata et al., 2019) (**Figure 5**; **Table 1**).

Salt stress disrupts plant physiology by inducing osmotic stress, ion toxicity, metabolic imbalance, and excessive ROS production (Zhao et al., 2021). Glycine betaine, with its low molecular weight, high solubility, and low viscosity, serves two primary functions to mitigate these effects. Acting as an osmoprotectant, glycine betaine stabilizes cytosolic proteins to prevent denaturation and serves as an osmolyte to balance the osmotic effects of Na^+^ and K^+^ accumulation in vacuoles, protecting cytoplasmic structures (Chen and Murata, 2011). Acting as a ROS scavenger, glycine betaine alleviates oxidative damage under salt stress, as demonstrated in common bean plants (Hasanuzzaman et al., 2014; Sofy et al., 2020). Therefore, glycine betaine mitigates salt stress through dual protective mechanisms: serving as an osmoprotectant to stabilize proteins and balance ionic osmotic pressure, while also functioning as an effective ROS scavenger to alleviate oxidative damage.

Glycine betaine responds to salt stress by regulating ion homeostasis. It influences ion homeostasis through modulating ion channels, reducing K^+^ efflux, and enhancing H^+^-ATPase activity in plasma membranes. In maize, glycine betaine has been shown to improve the expression of plasma membrane *H^+^-ATPases*, promote phosphate uptake, and regulate cellular Na^+^ and K^+^ levels under salt stress (Wei et al., 2017; Zhu et al., 2022). Exogenous application of glycine betaine to maize seedlings significantly enhanced antioxidant activity, ion balance, stress-tolerant gene expression, and photosynthesis, thereby improving their resistance to NaCl stress (Zhu et al., 2022; Islam et al., 2024). Under salt stress, glycine betaine enhanced the extrusion of sodium ions from maize roots and leaf protoplasts, thus improving the sodium-potassium balance. Additionally, glycine betaine significantly upregulated the expression of plasma membrane H^+^-ATPase genes *MHA2* and *MHA4*, as well as the Na^+^/H^+^ antiporter gene *NHX1*, resulting in enhanced salt tolerance (Zhu et al., 2022). Collectively, glycine betaine enhances plant salt tolerance by modulating ion channels and upregulating key genes (*MHA2*, *MHA4*, *NHX1*) to maintain cellular Na^+^/K^+^ homeostasis.

Glycine betaine plays a critical role in enhancing antioxidant defense by upregulating antioxidant enzymes such as *SOD*, *POD*, and *CAT*. It also enhances non-enzymatic antioxidants like proline and glutathione, reducing oxidative stress in vulnerable tissues. Glycine betaine contributes to membrane stabilization by maintaining thermodynamic protein stability, preventing protein aggregation, reducing lipid peroxidation, and protecting membrane permeability under saline conditions (Banu et al., 2010). Previous research demonstrates that exogenous glycine betaine improves salt tolerance in plants such as kidney beans by stabilizing photosystems, regulating membrane integrity, and maintaining cellular antioxidant defenses (Olmos et al., 2024). Additionally, glycine betaine protects the photosynthetic apparatus by shielding photosynthetic oxygen-evolving centers from oxidative damage and sustains Rubisco activity, mitigating photosynthetic reduction caused by salinity stress (Huang et al., 2020). Hence, glycine betaine enhances salt tolerance by strengthening antioxidant defenses through upregulation of both enzymatic (SOD, POD, CAT) and non-enzymatic (proline, glutathione) systems, while concurrently stabilizing membranes and protecting the photosynthetic apparatus from oxidative damage.

Glycine betaine also modulates endogenous phytohormones and gene expression in response to salt stress. For instance, studies have demonstrated that glycine betaine enhances ABA biosynthesis, thereby inducing stomatal closure to reduce transpiration (Chen and Murata, 2011). Additionally, under salt stress conditions, tobacco plants treated with glycine betaine exhibited a 5-fold upregulation of *BADH* gene expression, significantly improving salt tolerance (Sofy et al., 2020). These findings demonstrate that glycine betaine mediates plant adaptation to salt stress through coordinated mechanisms involving plant hormone signaling and gene regulation (**Figure 5**).

In summary, glycine betaine plays a central role in plant responses to salt stress. It functions not only as an osmoprotectant and ROS scavenger but also contributes to membrane stabilization, photosynthetic protection, and ion homeostasis. Through its regulatory effects on antioxidant defense systems, glycine betaine enhances plant resilience to salt stress, making it a key target for improving salt tolerance in crops. A comprehensive summary of glycine betaine biosynthesis and regulatory networks is presented in **Figure 5** and **Table 1**.

## 4 Concluding remarks and future perspectives

Significant progress has been made in understanding the roles of secondary metabolites such as flavonoids, melatonin, and glycine betaine in plant salt stress responses. However, several critical knowledge gaps remain, and future research should prioritize agricultural applications aimed at improving plant growth and yield under saline conditions.

Secondary metabolites frequently interact, introducing an additional layer of regulatory complexity. A key question is whether these inter-metabolite interactions directly modulate salt tolerance and, consequently, crop productivity. This is further complicated by the fact that many pathways share intermediate substrates, yet the dynamic flux of these metabolites under salt stress and its impact on yield-related traits remain largely unexplored. Addressing these questions requires experimental strategies that simultaneously perturb multiple pathways. To this end, physical interaction assays (e.g., Microscale Thermophoresis, Surface Plasmon Resonance) can be employed to detect direct binding between metabolites, while genetic interaction analyses, combined with metabolite feeding and complementation experiments, can uncover indirect functional relationships. Together, these approaches will provide critical evidence for synergistic effects among metabolites in enhancing stress adaptation and maintaining yield stability.

While significant progress has been made in understanding secondary metabolite functions at the molecular level, translating this knowledge into agricultural applications requires additional effort. Key priorities for improving plant growth and yield under salt stress include: (i) identifying natural genetic variation in biosynthetic and regulatory genes for marker-assisted breeding of high-yielding, salt-tolerant varieties; (ii) developing multiplex gene editing strategies to simultaneously enhance multiple metabolic pathways that contribute to stress resilience and yield maintenance; (iii) using tissue-specific or stress-inducible promoters to avoid unintended growth penalties while maximizing protective metabolite accumulation; (iv) applying synthetic biology approaches to reconstitute and optimize metabolic pathways for sustained crop performance in saline environments; and (v) integrating metabolite traits with other stress tolerance mechanisms (e.g., ion transporters, stress receptors) to achieve synergistic effects on plant growth and yield.

Plant secondary metabolism represents a highly versatile system for coping with salt stress, enabling plants to maintain homeostasis and achieve resilience under adverse conditions. Despite significant progress, the complex interplay between primary metabolism, secondary metabolism, and transcriptional regulation —and its collective impact on crop yield—remains incompletely understood. Future research should focus on elucidating how the modulation of secondary metabolite pathways can be translated into practical strategies for improving plant growth, biomass accumulation, and grain yield in salt-affected agricultural systems. These findings will ultimately aid in developing crop varieties with enhanced salt tolerance and sustained productivity, ensuring sustainable agricultural practices in saline-affected regions.
